# Humidity Sensor Based on Bragg Gratings Developed on the End Facet of an Optical Fiber by Sputtering of One Single Material

**DOI:** 10.3390/s17050991

**Published:** 2017-04-29

**Authors:** Joaquin Ascorbe, Jesus M. Corres, Francisco J. Arregui, Ignacio R. Matias

**Affiliations:** 1 Department of Electrical and Electronic Engineering, Public University of Navarra, Pamplona 31006, Spain; 2 Institute of Smart Cities, Public University of Navarra, Pamplona 31006, Spain; jmcorres@unavarra.es (J.M.C.); parregui@unavarra.es (F.J.A.); natxo@unavarra.es (I.R.M.)

**Keywords:** fiber optics sensors, diffraction gratings, transparent conductive coatings, thin-film, interference coatings, optical fiber humidity sensors

## Abstract

The refractive index of sputtered indium oxide nanocoatings has been altered just by changing the sputtering parameters, such as pressure. These induced changes have been exploited for the generation of a grating on the end facet of an optical fiber towards the development of wavelength-modulated optical fiber humidity sensors. A theoretical analysis has also been performed in order to study the different parameters involved in the fabrication of this optical structure and how they would affect the sensitivity of these devices. Experimental and theoretical results are in good agreement. A sensitivity of 150 pm/%RH was obtained for relative humidity changes from 20% to 60%. This kind of humidity sensors shows a maximum hysteresis of 1.3% relative humidity.

## 1. Introduction

Humidity has an important influence on several industrial processes, such as in electronics, food, or pharmaceutical manufacturing, as well as in food storage and others. All of these processes, which can be affected by humidity, force continuous monitoring the air humidity. Additionally, proper humidity levels can be critical to the quality of the final product and having the right humidity level can contribute to reducing energy consumption [1,2,3,4,5,6,7]. 

Optical fiber humidity sensors (OFHS) offer several advantages over electronic humidity sensors, such as miniature design, durability, the possibility of working in flammable environments and at higher temperature and pressure ranges and, most importantly, their electromagnetic immunity. Therefore, they can withstand the harsh and demanding conditions found in industrial processes. 

First, OFHSs utilized amplitude-based techniques [8,9,10,11,12], which measure changes on the transmitted optical power. This technique has a strong weakness as it depends on the power emitted by the light source, which can be altered by several undesired factors [13,14]. Nowadays, optical fiber sensors tend to use wavelength modulation methods to measure the target parameter because of its reliability with respect to the unpredictable fluctuations in optical power. 

Additionally, metal oxides have been previously proven as a good choice for the development of OFHS [10,15,16]. They can be coated onto an optical fiber by different methods, such as dip-coating [17,18,19] or sputtering [16,20]. Sputtering permits the fine-tuning of the sputtering conditions, which would allow obtaining denser nanocoatings, with lower porosity and greater refractive indices (RI) [21] when sputtering under high vacuum. Other parameters of the thin film could also be affected by the sputtering parameters as, for example, the crystallographic orientation, the degree of crystallinity, the carrier concentration, or even the stoichiometry [21,22,23]. 

Taking advantage of the versatility of the sputtering process, the development of a wavelength modulation device, such as a micro Bragg grating, on the end facet of a standard multimode optical fiber has been proposed in this paper as an OFHS. To our knowledge, this is the first time that a micro-grating has been built on the end facet of an optical fiber by using just one sputtering target, indium oxide (In_2_O_3_), and only changing the pressure of the sputtering process for humidity sensing purposes.

## 2. Materials and Methods 

### 2.1.Dependence of Indium Oxide Properties on the Vacuum Pressure of Sputtering

In this section some properties of sputtered indium oxide are analyzed, studying the influence of the sputtering parameters on the thin-film obtained. For this study, indium oxide was sputtered at high (P < 2 × 10^−5^ mbar) and low vacuum (P = 1.5 × 10^−1^ mbar) using a pulsed DC sputtering system from Nadetech Innovations (Pamplona, Spain).

**Figure 1:**
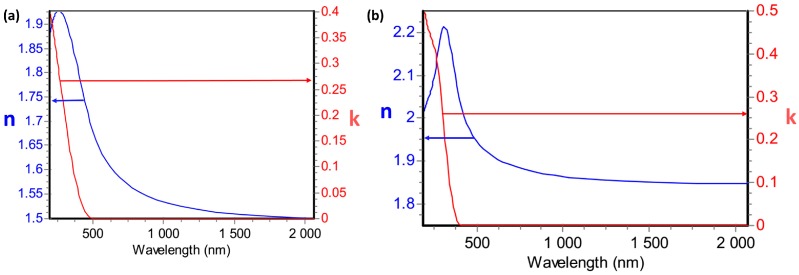
Optical constants of indium oxide sputtered at (**a**) low vacuum and (**b**) high vacuum.

The first parameter that was studied was the RI of indium oxide. RI is a complex number consisting of a real part (n) and an imaginary part (k) or extinction coefficient. Both components of the RI can be determined by ellipsometry measurements, which have been done using Uvisel-2 ellipsometer from Horiba (Kyoto, Japan). In Figure 1, n and k are plotted, as a function of the wavelength, for high and low vacuum conditions.

Figure 1 shows an almost constant variation of the real part of the RI of In_2_O_3_. Greater values of n are obtained when sputtering at high vacuum (12% greater). The value of n can be taken as a constant for long wavelengths and it is 1.83 for high vacuum sputtering, whereas this value decreases until 1.5 when sputtering at low vacuum. In a similar way, the extinction coefficient is greater when sputtering at lower pressures. 

Measurements of the roughness and the deposition rates have also been performed. Roughness was estimated by the scanning probe microscope (SPM) Innova from Veeco (New York, NY, USA). There are differences on the roughness of indium oxide depending on the sputtering conditions. Indium oxide sputtered at low vacuum has a RMS roughness of 12.3 nm, whereas the roughness is just 4.13 nm for high vacuum conditions. Thicknesses of deposited thin-films were estimated by the SPM and also by a quartz crystal microbalance (QCM). The deposition rate for low vacuum sputtering was 140 nm/min, whereas the deposition rate for high vacuum sputtering ranges from 250 to 40 nm/min, showing a high dependence on the position with respect to the cathode.

### 2.2. Device Fabrication

These OFHS were fabricated by coating the end facet of a standard communications multimode optical fiber (MMF) (optical fibers from Telnet RI, Zaragoza, Spain), by sputtering with an indium oxide (In_2_O_3_) target purchased from ZhongNuo Advanced Material Technology Co. (Beijing, China). For the development of the grating it is necessary to have different RIs for each layer. This requirement can be satisfied using only one material due to the dependence of the RI on the sputtering parameters. When coating with indium oxide, low refractive index layers (LRIL) have been obtained sputtering at high pressures, whereas high refractive index layers (HRIL) have been obtained at high vacuum (lower pressure).

For this purpose, an on/off control was applied to the valve that regulates the argon flow. The sputtering system was controlled on constant power mode (30 W). Therefore, the discharge voltage and sputtering current are auto-set as a function of the vacuum chamber pressure in order to maintain the sputtering power. 

Three different devices (D1–D3) with different thicknesses have been fabricated. Changes on the thickness of each period enable to tune the wavelength of the reflected peak. Parameters used for the development of these devices are commented in Table 1. Thicknesses of each layer were estimated using a quartz crystal microbalance (QCM) and, subsequently, those values are corroborated or corrected by comparison of the simulated results. Similarly, optical constants were measured by ellipsometry for both extreme sputtering conditions (low and high vacuum) and afterwards, they were compared with the data obtained by the theoretical analysis in order to obtain the correspondent value for each process. The devices D1, D2, D3, D4 and D5 have 60, 50, 24, 38 and 19 periods, respectively, where a period is formed by a pair of lower refractive index layer (odd layer) and a higher refractive index layer (even layer). More details about the fabrication process can be found in the literature [18].

### 2.3. Experimental Setup

**Figure 2:**
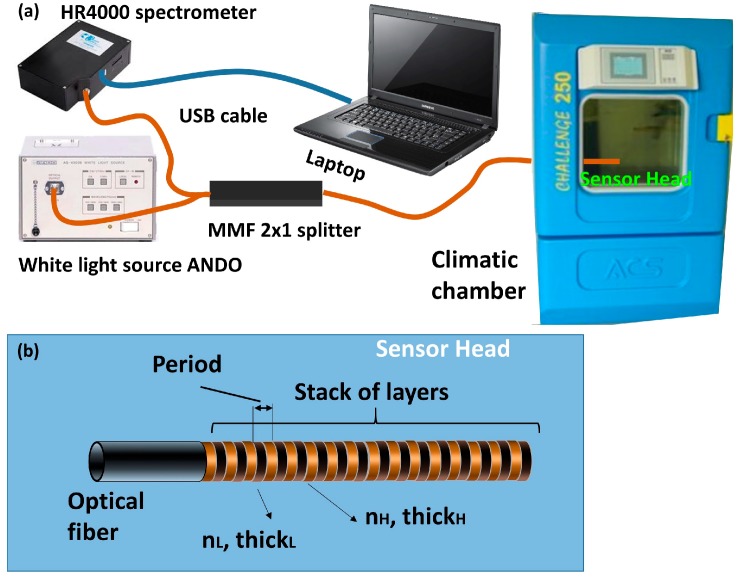
(**a**) Experimental setup for the characterization of the OFHS, and (**b**) a representation of the sensor head.

Standard communication MMF with a 62.5/125 µm core and cladding diameters, respectively, were used for the development of the OFHS. MMF enables easy coupling of the light launched by a white halogen light source (ANDO AQ-4303B). Light was launched through a 2 × 1 (50:50) splitter in a reflection setup. The other arm of the splitter was connected to an optical spectrometer (HR4000 from OceanOptics), which collects the light reflected from the end facet of a MMF pigtail with the grating on it (Figure 2b). This pigtail was introduced in a climatic chamber (ACS CH 250 from Angelantoni Industries) where several cycles of 20% to 60% relative humidity (RH) were applied while the temperature was kept constant to 25 °C. The RH started at 20%RH and it was increased with a slope of 1.33%RH/min until reaching 60%RH. The same parameters were used for decreasing the RH and there was also an addition final step to stabilize when it reached 20%RH. The experimental setup, and a scheme of the optical structure, are shown in Figure 2.

## 3. Results

### 3.1. Theoretical Analysis

In this section a numerical method based on plane wave propagation in a one-dimensional stack of layers of different RIs [24,25] has been followed in order to obtain some simulated results. Simulations based on the rigorous coupled-wave analysis, which gives as a result a set of four coupled-wave equations, expressed with a coupling matrix that relates the components of both magnetic and electric fields at both sides of each layer, show the main factors that might affect the sensitivity of this optical structure and its performance as an OFHS. These simulated results were obtained using Matlab^®^. Each reflection spectrum with a stack of layers was normalized with respect to the reflection spectrum without a stack of layers. Dispersion of RI of the material was not taken into account in order to simplify the analysis of the obtained results. Simulated results were made taking into account the dispersion of the refractive indices, showing changes in the obtained reflected spectra. However, these changes are related to the shape of the reflected peak and sidelobes, but they do not affect to the general behavior of the optical structure. The main factors influencing this optical structure are: number of periods, thickness, and RI of each layer.

For the correspondent simulations the RI of the core of the optical fiber was set to 1.5, according to the data obtained from Telnet-RI, while the RI of the external medium was set to 1. The effect of the water adsorbed onto the indium oxide layer can be modelled by the following phenomena: due to the polar nature of the water molecule, electrostatic attraction between the oxygen of the water molecule and the cationic side of the metal oxide surface occurs [26]. Therefore, this initial monolayer of water is generally chemisorbed [27]. Additional layers of water molecules start to be formed on the chemisorbed one and many more physisorbed layers will be joined for higher humidity values [26,27]. Therefore, the real part of RI (n) for LRIL was set to 1.7 and the extinction coefficient (k) was set to 0.0256 for the lowest humidity values (case A of Table 2) and n of LRIL was set to 1.8 and k to 0.0271 for the highest humidity values (case F of Table 2). Optical constants of HRIL have been kept as 4% greater than LRIL. These initial values of RIs have been chosen to agree with previous research [16,22,23,28]. Finally, a thin-film layer of water (50 nm of a layer with n = 1.33) has been added when simulating high RH values to simulate the layer of water physisorbed onto the coating surface. The particularities of the parameters for each situation under study are commented in their respective section and the general parameters are shown in Table 2.

These parameters allow to simulate the behavior of this optical structure for variations in RH. An increase of the RH implies an increase of the RI of the coating, which leads to a redshift of the reflected peak. Reflected optical power decreases as a result of the layer of water physisorbed on the coating.

#### 3.1.1. Number of Periods

**Figure 3:**
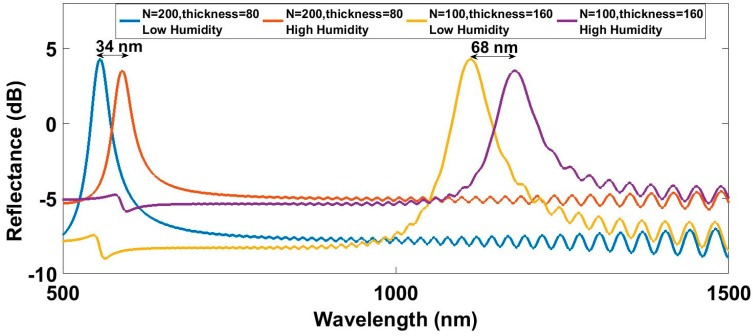
Simulated reflected spectra for extreme RH values with different number of periods but equal overall thickness.

First, it has been analyzed how the number of periods and the overall thickness affect the sensitivity. The overall thickness has been maintained constant, having two different simulated devices: the first one with 200 layers of 80 nm and the other one with 100 layers of 160 nm. The simulated spectra are shown in Figure 3.

The device with greater number of periods of smaller thickness has its reflected peak located at 561 nm and it shows a wavelength shift of 34 nm, while the device with 100 layers of 160 nm each one has its reflected peak located at 1122 nm and presents a wavelength shift of 68 nm. The previous figure shows greater sensitivities for reflected peaks located at greater wavelengths. The overall thickness of the coating and the number of periods do not influence the sensitivity, although it might increase the response time. The next analysis is focused on finding how this relationship between wavelength and sensitivity relates.

#### 3.1.2. Thickness of Layers

**Figure 4:**
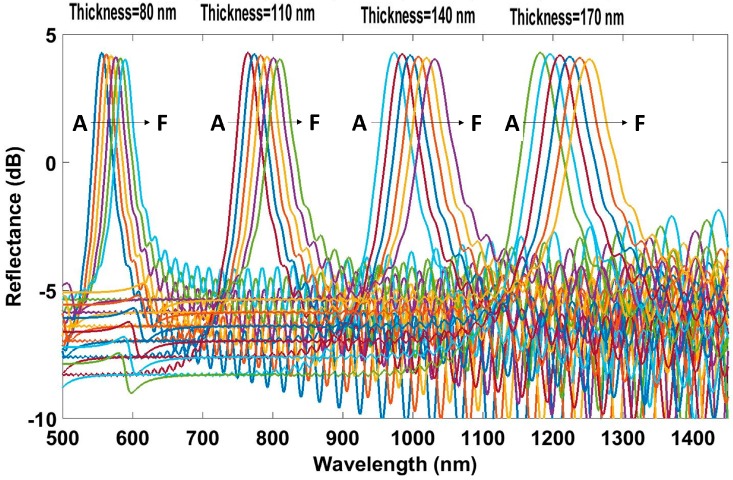
Simulated humidity effect on the reflected spectra for different thicknesses of periods.

The tuning of the reflection wavelength, given a specific material, is achieved changing the thickness of each layer. In order to study the influence of the thicknesses of the lower and higher refractive index layers on the humidity sensitivity, the following parameters were used: the number of periods was set to 50 and the thickness of odd and even layers was swept from 80 to 170 nm in 30 nm intervals. The simulated results are shown in Figure 4.

As can be observed, the thickness of each layer influences the width of the reflected peak. For a fixed difference in the RIs of the odd and the even layers, the width of the reflected peak increases if the thickness of both layers is increased. However, the most important effect is related to the sensitivity. The wavelength displacement is greater for thicker layers. This is inherent to the operation of this optical structure [29,30]. The simulated results show how the wavelength displacement depends on the wavelength where the reflected peak is located. 

#### 3.1.3. Refractive Index

**Figure 5:**
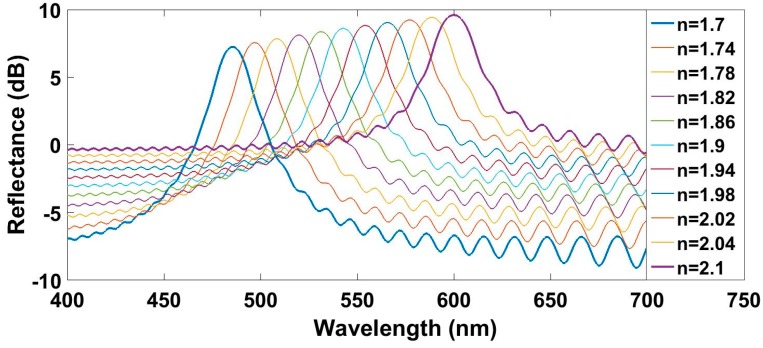
Simulated reflected spectra for different refractive indices with a fixed thickness of 70 nm.

The last factor analyzed is the RI of the layers composing the grating. In order to obtain a fair comparison, the ratio between the RIs of the even layers and the odd layers was always kept constant and equal to the ratio used in previous simulations, and the sensitivity obtained is analyzed as a function of the reflection wavelength. RI of LRIL was swept from 1.7 to 2.3, while RI of HRIL was always maintained 4% greater. In Figure 5, the obtained spectra for different refractive indices and a fixed thickness for each layer of 70 nm are plotted. The legend of Figure 5 refers to the RI of the LRIL. It can be seen that an increasing difference between the RI of the optical fiber and the material lead to lower contrast. Reflected spectra with a worse-defined peak might difficult the performance of the processing algorithm.

The previous figure shows contrast decreasing from 14 dB for a real part of the RI of the LRIL of 1.7 to less than 10 dB when that RI has a value of 2.1. However, the reflected power increases at all wavelengths for greater real parts of the RI. The reflected peak widens with an increase of the real part of the RI. 

**Figure 6:**
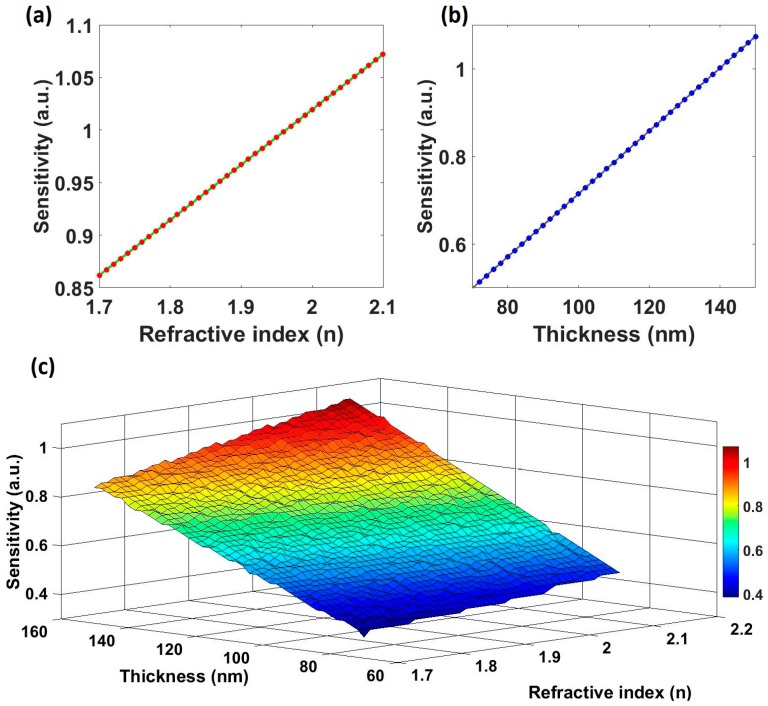
(**a**) Sensitivity as a function of the reflection wavelength achieved by different RI of the material, (**b**) evolution of the sensitivity as a function of the thickness of each layer and (**c**) representation of the dependence of the sensitivity of this optical structure on thickness and RI in a surface plot.

Figure 6 shows the linear dependence of the sensitivity with respect to (a) the RI and (b) the thickness. In Figure 6c a 3D graph representing the sensitivity as a function of the RI and the thickness is plotted. The sensitivity increases with the RI of the material, but its dependence is weaker than the one obtained for the thickness, due to its lower influence on the optical path. That means that it is easier to place the reflection peak at greater wavelengths and, consequently, obtaining greater sensitivities by increasing the thickness than by increasing the RI.

### 3.2 Experimental Results

#### 3.2.1. Relative Humidity Measurements

**Figure 7:**
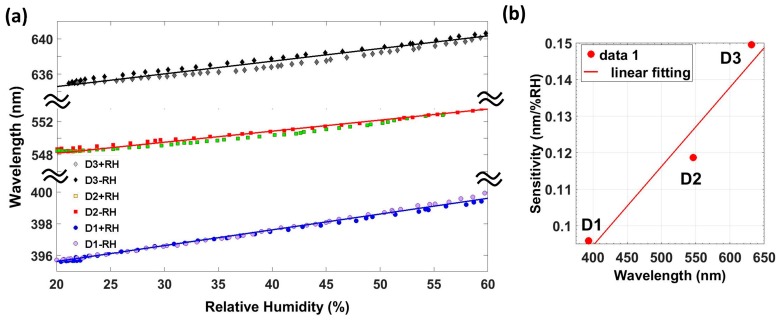
(**a**) Reflection wavelength as a function of the relative humidity for the three devices and (**b**) sensitivity as a function of the reflection wavelength for relative humidity ranging from 20%RH to 60%RH.

Here the performance of devices D1 to D3 is discussed. Device D1 consists of 60 periods of 115 nm each, which leads to obtaining a reflected peak at 390 nm. Device D2 consists of 50 periods of 162 nm, which has its reflected peak located at 550 nm, and the last device analyzed (D3) consists of 24 periods, has its reflected peak located at 635 nm, and has an overall thickness of 4.4 µm. This device has the smaller overall thickness, but the optical path of each period results in the reflected peak being located at greater wavelengths. In Figure 7a, a comparison of the wavelength shift of the three devices developed on the 20–60%RH is shown. The hysteresis for this RH range is 1.3%RH. Finally, the sensitivity of these devices is plotted as a function of the reflection wavelength in Figure 7b. 

**Figure 8:**
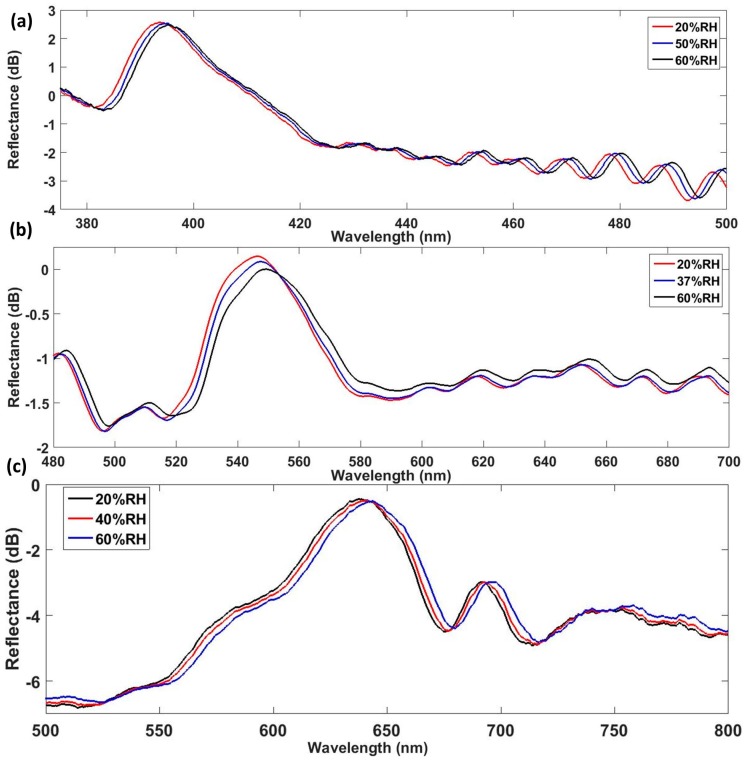
Measured reflected spectra for different RH values obtained with (**a**) device D1, (**b**) device D2, and (**c**) device D3.

As it can be observed in Figure 7b, the sensitivity of this optical structure increases with the Bragg wavelength. It means that, for a specific material with its own RIs, greater sensitivities will be achieved by increasing the length of the periods. The spectrometer used has a resolution of 0.26 nm. Taking into account the maximum sensitivity obtained (0.15 nm/% RH), this optical structure enables to measure RH with a resolution of 1.7%RH. Figure 8 depicts the measured reflected spectrum obtained with the three devices for different values of RH. All devices show the same behavior as humidity increases. Reflection wavelength redshifts and the reflected optical power decreases as the RH increases, due to the layer of water physisorbed onto the coating surface.

**Figure 9:**
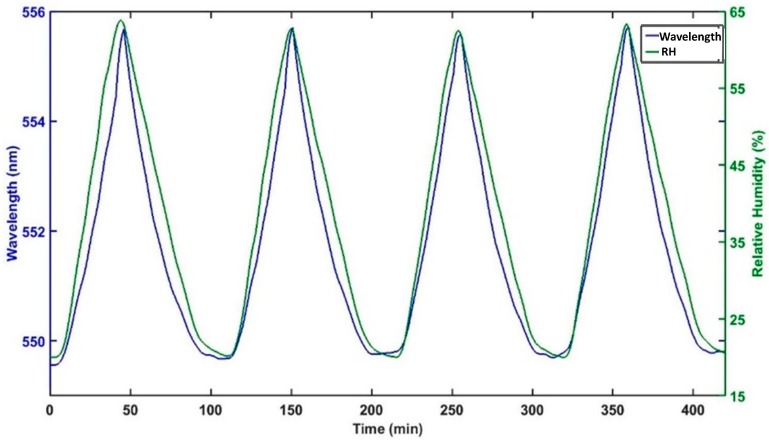
Measured reflected wavelength and RH plotted as a function of time for device D2.

Figure 9 shows continuous monitoring of the reflected spectrum and its correspondent relative humidity in order to give a better view of the dynamic response of this kind of device. The evolution of the reflected wavelength and the relative humidity as a function of time for device D2 is represented. The reflection wavelength was calculated by a simple algorithm, which, at first, calculates the maximum reflected power and the wavelength where it occurs. Then, the algorithm approximates the reflected peak by a parabola and locates the vertex of the parabola. It can be seen that the reflected peak redshifts when the relative humidity increases and returns to its original wavelength when the RH decreases until 20%RH.

#### 3.2.2. Influence of Temperature

**Figure 10:**
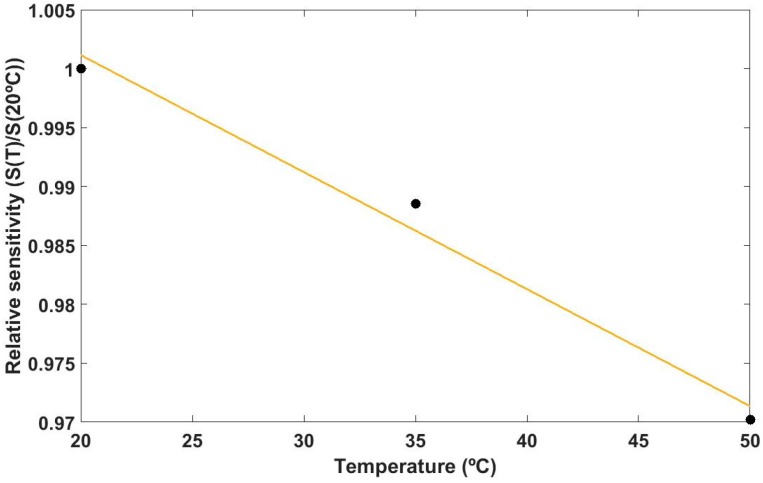
Relative sensitivity as a function of the temperature.

Device 4 was used for the study of the influence of the temperature. It consists of a grating of 38 periods. This experiment was carried out to examine how the temperature might affect to the sensitivity of the device. Device 4 was subjected to 20–60%RH cycles at three different temperatures: 20, 35, and 50 °C.The sensitivity to RH decreases at a 1 × 10^−3^ (nm/%RH)/°C, as depicted in Figure 10. 

**Figure 11:**
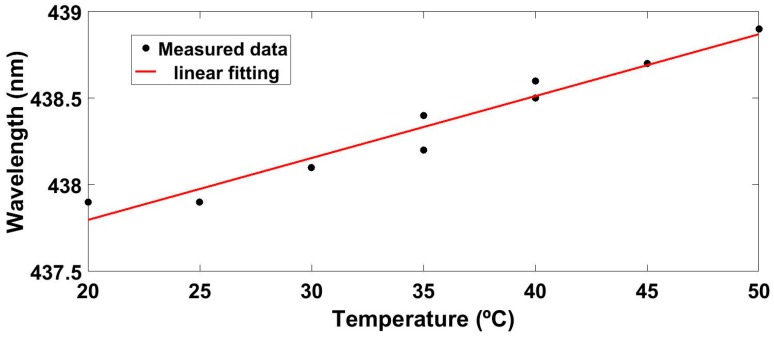
Sensitivity of the optical structure to temperature.

The sensitivity of the device to temperature has also been studied and for this purpose the same experimental setup of Figure 2 was used. The temperature was changed from 20 to 50 °C in 5 °C steps, while RH was kept constant at 20%RH and the measured results are shown in Figure 11. As it can be seen, the Bragg wavelength shifts 1 nm to greater wavelengths as the temperature increases from 20 to 50 °C. This response is linear as it can be observed in Figure 11. This optical structure has a sensitivity of 0.036 nm/°C, which leads to a cross thermal sensitivity of 0.36%RH/°C and to a linear coefficient of thermal expansion of 7.6 × 10^−5^ (°C)^−1^.

#### 3.2.3. Response Time

**Figure 12:**
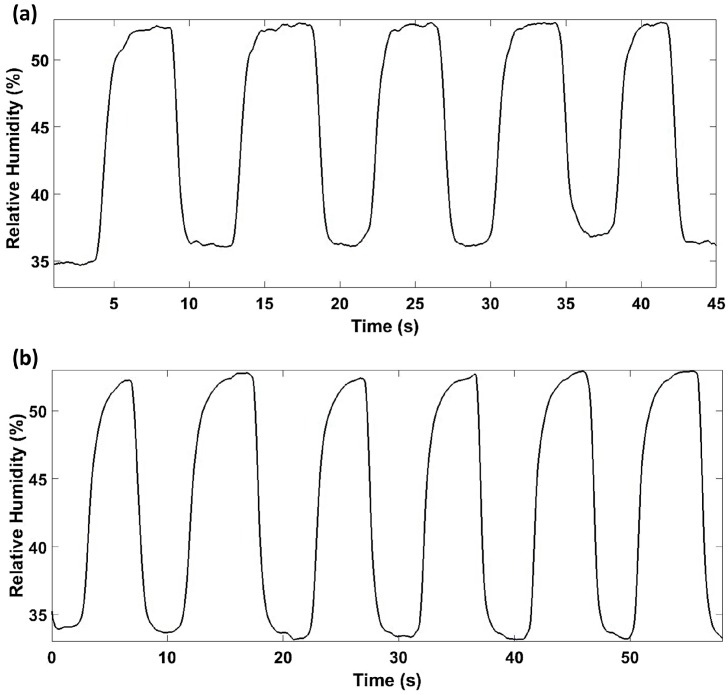
Response time of the optical device with (**a**) 19 periods, and (**b**) 38 periods.

Finally, the response time of the device has been studied. Two different devices, devices 4 and 5, were analyzed to compare the effect of the number of periods. It was necessary to change the setup in order to measure the response time due to the relatively slow slope of the climatic chamber. Therefore, changes on the relative humidity were produced by using different salt solutions, such as magnesium chloride, which provides a constant RH environment of 33%RH, and magnesium nitrate, which provides a controlled environment at 53%RH. The two different optical fiber humidity sensors were alternatively immersed in these environments. Figure 12 shows the dynamic response of both devices. The rise time is 1.9 s, whereas the fall time is 1.4 s. There is no noticeable differences between both devices. The fact that the value of the rise time is greater is attributed to the method of the measurement, since desorption time is usually greater.

## 4. Discussion and Conclusions

An optical structure consisting of a grating generated on the end facet of standard multimode optical has been fabricated and its performance as a humidity sensor has been studied. The grating was fabricated with only one material, indium oxide (In_2_O_3_), by means of sputtering. Differences on the RI of indium oxide, needed for obtaining the grating, were achieved by changing the sputtering pressure. A theoretical analysis, based on the humidity sensing mechanism of metal oxides, has been made to check how the different parameters involved in the fabrication of these optical devices affect the sensitivity of the sensor. The reflection wavelength obtained with this structure can be tuned in a range of interest, enabling measurements at the whole ultraviolet-visible-near-infrared (UV-VIS-NIR) range offered by commercially-available spectrometers.

Simulated and experimental results are in good agreement with regard to the behavior of this structure. The main factor affecting the sensitivity of this kind of devices is the thickness of each layer or period. Total thickness does not seem to have great influence in sensitivity. The sensitivity of this optical structure depends on the reflection wavelength (the longer the reflection wavelength the higher the sensitivity) and the maximum sensitivity obtained was 150 pm/%RH for relative humidity ranging from 20% to 60% with device D3 (device with less periods which are thicker). The sensitivity follows a linear relationship with the reflection wavelength. These OFHSs show good repeatability and low hysteresis. The maximum resolution of this optical structure is 1.7%RH.

With regard to the influence of temperature on the performance of this kind of devices the following Measured sensitivity to temperature is 0.036 nm/°C, which leads to a cross-thermal sensitivity of 0.36%RH/°C. The sensitivity follows a linear relationship with the reflection wavelength.

Further research should be conducted in order to check if the number of periods has some influence on the amount of water adsorbed. Greater number of periods might difficult water to penetrate to the initial layers of the material, decreasing the sensitivity of the device, and the simulated results do not allow taking into account these physical effects. This study can be extended to other substances, such as volatile organic compounds. 

This new method can be extended to co-sputtering, reactive sputtering, or any physical vapor deposition method. Co-sputtering can lead to the development of a multiple parameter sensing device. Additionally, this structure is expected to enable the fabrication of optical sensors for several purposes, such as biosensors, chemical sensors, or gas detection. 

## Figures and Tables

**Table 1:** Sputtering parameters for the fabrication of the optical fiber humidity sensors.

	N ^1^	Layer	P (mbar)	V (Volts)	I (mA)	Time (s)	Thickness (nm)	λ_B_ (nm)
**D1**	60	Odd	8 × 10^−2^	186	160	30	64	390
		Even	2 × 10^−5^	353	80	40		
**D2**	50	Odd	1 × 10^−1^	153	200	36	83	550
		Even	2 × 10^−5^	237	150		79	
**D3**	24	Odd	1.5 × 10^−1^	160	150	60	92	635
		Even	2 × 10^−5^	278	100	80		
**D4**	38	Odd	7 × 10^−2^	169	180	30	71	432
		Even	2 × 10^−5^	381	76	40		
**D5**	19	Odd	4 × 10^−2^	172	190	36	69	454
		Even	2 × 10^−5^	330	100		74	

^1^ N refers to the number of periods.

**Table 2:** Simulation parameters for the study of the effect of humidity on indium oxide.

	n_LRIL_	k_LRIL_	n_HRIL_	k_HRIL_	Water Layer (nm)
**A**	1.70	0.0256	1.7680	0.0266	0
**B**	1.72	0.0259	1.7888	0.0269	10
**C**	1.74	0.0262	1.8096	0.0272	20
**D**	1.76	0.0265	1.8304	0.0275	30
**E**	1.78	0.0268	1.8512	0.0278	40
**F**	1.80	0.0271	1.8720	0.0281	50
